# Potential therapeutic role of punicalagin against mechanical-trauma-induced stress urinary incontinence via upregulation of Nrf2 and TGF-β1 signaling: Effect of punicalagin on mechanical trauma induced SUI

**DOI:** 10.1007/s00192-017-3283-x

**Published:** 2017-02-06

**Authors:** Jianming Tang, Cheng Liu, Jie Min, Ming Hu, Yang Li, Li Hong

**Affiliations:** Department of Gynecology and Obstetrics, Renmin Hospital of Wuhan University, #238 Liberation Road, Wuhan, 430060 Hubei Province People’s Republic of China

**Keywords:** Mechanical trauma, Vaginal distension, Punicalagin, Stress urinary incontinence, Oxidative damage, Extracellular matrix

## Abstract

**Introduction and hypothesis:**

We investigated the effect of punicalagin (PUN; 2,3-hexahydroxydiphenoyl-gallagyl-D-glucose), on mechanical-trauma-induced stress urinary incontinence (SUI) in mouse and the mechanisms underlying any effects.

**Methods:**

Ninety virgin female C57BL/6 mice were randomized into six groups: five groups underwent vaginal distention (VD) for 1 h and leak-point pressure (LPP) was measured on the 1st, 3rd, 7th, 14th, and 28th day following (VD groups 1 d, 3 d, 7 d, 14 d, and 28 d). The sixth group was a noninstrumented control (NC) group. Then, 75 virgin female C57BL/6 mice were randomized into five groups: a VD group (that just underwent VD) and an NC group were orally administered saline every day for 7 days; and three VD + PUN groups that underwent VD and were orally administered PUN respectively at 2.5, 5, and 10 mg/kg every day for 7 days. LPP was tested on the day 7, then all mice were sacrificed and their urethras and anterior vaginal walls harvested for Masson staining, immunohistochemistry study, Western blot analysis, and quantitative polymerase chain reaction (qPCR).

**Results:**

LPPs after VD were significantly lower than the NC group, and the LPPs of mice on days 14 and 28 day after VD were significantly higher than on the days 1, 3, and 7. PUN significantly improved VD-induced drops in LPP and alleviated VD-induced decrease of collagen I, collagen III, α-smooth muscle actin (SMA), transforming growth factor (TGF)-β1, and p-Smad3, nuclear factor-erythroid 2 p45-related factor 2 (Nrf2), and glutathione peroxidase (GPx1) protein levels, and increase of 8-hydroxydeoxyguanosine (OHdG) in urethra and anterior vaginal wall. PUN also up-regulated the expression of manganese superoxide dismutase (MnSOD), whereas protein levels of Smad 2, p-Smad2, and Smad3 were not changed.

**Conclusions:**

PUN exerts certain therapeutic effect on mechanical-trauma-induced SUI in mice, which might be through the activation of TGF-β1/Smad3 and Nrf2/antioxidant response element (ARE) signaling activation.

## Introduction

Stress urinary incontinence (SUI) is a common social and hygiene problem affecting the quality of life (QoL) of 25~57% adult women worldwide and causing serious social economic load [1–5]. Although progress has been made in treating SUI in recent decades, pharmacological therapy is poorly understood. Therefore, drug discovery and development for SUI is extremely urgent. The etiologic and pathophysiologic mechanisms, however, have not been well elucidated. Aging, vaginal childbirth, declined hormonal status (menopause), and obesity were the main risk factors of SUI [4–6]. Increasing research confirmed that mechanical-trauma-induced oxidative damage and extracellular matrix (ECM) remodeling are probably involved in the pathogenesis of SUI, especially birth trauma and increased abdominal-pressure-induced SUI or pelvic organ prolapase (POP) with SUI [7–11]. Nuclear factor-erythroid 2 p45-related factor 2 (Nrf2) is an upstream transcription factor modulating antioxidant ability [12]. Upon oxidative stress, Nrf2 parts from kelch-like ECH-associated protein 1 (Keap1) and translocates into the nucleus to induce the expression of antioxidant response elements (ARE), such as glutathione peroxidase (GPx), superoxide dismutase (SOD), catalase (CAT), and heme oxygenase (HO)-1, against oxidative damage [12]. Collagen is an important component in ECM and plays a critical role in maintaining the normal functions of the pelvic support structure. Previous studies demonstrated that incontinence is associated with reduced content of collagen I, III, and α-smooth muscle actin (SMA) [6, 13–17]. Transforming growth factor β (TGF-β), including TGF-β1, TGF-β2, and TGF-β3, a pleiotropic cytokine, plays important roles in many physiological and pathological processes; in mammals, the primary factor is TGF-β1, and the TGF-β1/Smads pathway plays an important role in the regulation of collagen metabolism.

Accrding to the Chinese* Compendium of Materia Medica*, pomegranate peel is used to treat archoptoma, dysentery, hematockezia, morbid leucorrhoea, uterine bleeding, and uroclepsia. Punicalagin (2,3-hexahydroxydiphenoyl-gallagyl-D-glucose; PUN), the major bioactive component of pomegranate peel, has antioxidant, anti-inflammatory, antiviral, antiapoptosis, and anti-collagen-degradation properties [18–20]. Previous studies demonstrated that PUN inhibits lipopolysaccharide (LPS)-induced oxidative stress via upregulation of the Nrf2/HO-1 pathway and alleviates oxidative damage [18]. Additionally, PUN shows anti-collagen-degradation activity in vitro [20]. In the work reported here, we tested the hypothesis that PUN plays a role in treating mechanical-stress-induced oxidative damage and ECM remodeling, even the potential therapeutical effect on mechanical-trauma-induced SUI.

## Materials and methods

### Reagents

Punicalagin [>98% high-performance liquid chromatography (HPLC) purity] was purchased from Chengdu must Bio-tech (Chengdu, China). Antibodies to β-actin (ab8227), collagen I (ab21286), collagen III (ab7778), α-SMA (ab124964), Nrf2 (ab137550), TGF-β1 (ab92486), and GPx1 (ab22604) were obtained from Abcam (Cambridge, UK). Antibodies to Smad2 (5339p), Smad3 (9523p), p-Smad2 (3108p), and p-Smad3 (9520p) were purchased from Cell Signaling Technology (Danvers, MA, USA). Antibody to MnSOD (06–984) was purchased from Millipore (Billerica, MA, USA). Fluorescence-labeled secondary antibodies (IRDye700 and IRDye800, goat antimouse/rabbit) was purchased from Licor, Inc. (Lincoln, NE, USA).

### Experimental animals and study design

Leak-point pressure (LPP) reflects the ability of urinary insistence, hence the LPPs of mice in every group were measured to determine the identification index of SUI in mice. In the first part of this research, in order to investigate the efficiency and duration of a vaginal distention (VD)-induced mouse model of SUI, 90 wild-type virgin female C57BL/6 mice (8~10 weeks old) were randomized into six groups: a noninstrumented control (NC) group; and five groups underwent VD for 1 h with 8-mm dilators (0.3 ml saline) and their LPPs were measured respectively on days 1, 3, 7, 14, and 28 after VD (groups VD 1 d, 3 d, 7 d, 14 d, and 28 d). There was no statistically significant difference among body weights of mice between groups (Table 1). In the second part of this research, to explore the potential therapeutic role of PUN against VD-induced SUI and its underlying mechanism, 75 wild-type virgin female C57BL/6 mice (8~10 weeks old) were randomized into five groups: NC, VD, and VD + PUN 2.5, 5, and 10. VD and VD + PUN 2.5, 5, and 10 mice underwent VD for 1 h with 8-mm dilators the first day, then the VD + PUN 2.5, 5, and 10 groups were orally administered 2.5, 5, and 10 mg/kg of PUN, respectively, every 24 h during the entire experimental period. Mice in the NC and VD groups were fed with equal amount of 0.9% normal saline. LPPs of mice in all groups were measured on the day 7 after VD. Animals were sacrificed, and urethras and anterior vaginal walls were harvested for immunohistochemistry study, Western blot analysis, and quantitative polymerase chain reaction (qPCR). There was no statistically significant difference among body weight (Table 2). All experimental protocols were approved by the Institutional Animal Care and Use Committee of Renmin Hospital of Wuhan University.

**Table 1:** Body weights of mice in six groups $\left(\overline{x}\pm S\right)$

Group	Weight (g)	*F* value	*P* value
NC	16.04 ± 0.71	0.36	0.73
VD 1 d	16.20 ± 0.64		
VD 3 d	15.97 ± 0.66		
VD 7 d	16.23 ± 0.50		
VD 14 d	16.05 ± 0.78		
VD 28 d	16.17 ± 0.70		

*NC* noninstrumented control,* VD* vaginal distention,

**Table 2:** Body weights of mice in five groups $\left(\overline{x}\pm S\right)$

Group	Weight (g)	*F* value	*P* value
NC	16.10 ± 0.73	0.20	0.99
VD	16.03 ± 0.72		
VD + PUN 2.5	15.97 ± 0.70		
VD + PUN 5	16.13 ± 0.64		
VD + PUN 10	15.95 ± 0.67		

*NC* noninstrumented control,* VD* vaginal distention, *PUN* punicalagin

### Vaginal distention

Mice in the experimental groups underwent VD after being anesthetized with urethane (1 g/kg, i.p.). After lubrication with paraffin oil, a modified 6-F Foley catheter was inserted into the vagina and secured to the vaginal introitus with a 5/0 silk suture. Then, 0.3 ml distilled water was infused into the balloon to distend the vagina. Each balloon’s diameter was measured before VD using a Vernier caliper. After 1 h, the balloon was deflated and removed, and the mouse permitted to wake spontaneously. The NC group did not undergo VD.

### Suprapubic tube implantation and LPP measurement

One day before LPP measurement, a epidural catheter was implanted in the bladder under urethane (1 g/kg, i.p.) anesthesia. On the day of LPP measurement, mice were again anesthetized with urethane (1 g/kg, i.p.), the bladder catheter was connected to both a micro syringe pump and a pressure transducer of a urinary dynamics detector (Nidoc970C, Weixin Medical of China) through a T-branch pipe. Pressure and force transducer signals were amplified and digitized for computer data collection. The bladder was then filled with room-temperature saline at 1 ml/h through the bladder catheter. When half the bladder capacity was reached, gentle pressure with one finger was applied to the mouse’s abdomen. Pressure was gently increased until urine leaked, at which time the externally applied pressure was rapidly removed. Peak bladder pressure was used as the LPP. Voids could be easily distinguished from leaks. If a mouse voided, the bladder was refilled and the process was repeated. At least five LPPs were obtained on each animal and the mean calculated.

### Western blot

Total protein was extracted from specimens using radioimmunoprecipitation assay (RIPA) buffer containing phenylmethylsulfonyl fluoride (PMSF). Proteins were denatured at 95 °C after concentration measurement, then 30 μg of the total protein was separated from these samples by 10% sodium dodecyl sulfate (SDS)–polyacrylamide gel electrophoresis (PAGE) then transferred onto polyvinylidene fluoride (PVDF) membranes. After being blocked, membranes were sequential blotted with primary and secondary antibodies (1:10000). Signals were detected with an Odyssey infrared imaging system (LI-COR Bio, USA). Primary antibodies were as follows: anti-Nrf2 (1:500), anti-GPx1 (1:500), anti-MnSOD (1:500), anti-TGF-β1 (1:250), anti-Smad2 (1:250), anti-p-Smad2 (1:250), anti-Smad3 (1:250), anti-p-Smad3 (1:250), anticollagen I (1:100), anticollagen III (1:250), anti-α-SMA (1:5000), and anti-tissue inhibitor of metalloprotease (anti-TIMP-3) (1:1000).

### Quantitative real-time polymerase chain reaction

Primers were purchased from Sangon Biotech (Shanghai, China). Total RNA was extracted using RNAiso Plus (TaKaRa Biotech, Dalian, China), and first-strand complementary (c) DNA was synthesized using a PrimeScript^TM^ RT regent Kit (TaKaRa Biotech, Dalian, China). q-PCR was conducted using SYBR^R^ Premix Ex Taq^™^ II Kit (TaKaRa Biotech, Dalian, China). SYBR green real-time PCR mix for PCR containing 7.5 μM each of forward and reverse primers (listed in Table 3). The reaction conditions were as follows: predenaturation at 95 °C for 30 s; 40 cycles of 95 °C for 5 s, and 60 °C for 34 s; then a final extension stage of 95 °C for 15 s, 60 °C for 1 min, and 95 °C for 15 s. β-actin was used as the reference gene. Relative quantification of gene expression for both target and reference genes was performed by the 2^-ΔΔCt^ method and based on Ct values. Real-time PCR analysis results are presented as mean ± standard deviation (SD) of fold change in expression.

**Table 3:** Primers for quantitative real-time polymerase chain reaction

Gene name	Gene ID	Primer sequence (5′-3′)	Amplicon size (bp)
Collagen I (A1)	NM_007742.3	F: AAGAAGCACGTCTGGTTTGGAG	175
		R: GGTCCATGTAGGCTACGCTGTT	
Collagen III (A1)	NM_009930	F: GTGGCAATGTAAAGAAGTCTCTGAAG	191
		R: GGGTGCGATATCTATGATGGGTAG	
α-SMA	NM_031004.2	F: AACTGGTATTGTGCTGGACTCTG	172
		R: CTCAGCAGTAGTCACGAAGGAATA	
β-actin	NM_007393.3	F: GTGACGTTGACATCCGTAAAGA	287
		R: GTAACAGTCCGCCTAGAAGCAC	

*SMA* smooth muscle actin

### Immunohistochemistry and Masson staining

All specimens were embedded in paraffin and cut into 4-μm-thick slices and fixed to glass slides. For immunohistochemical staining, sections were deparaffinized in xylene and rehydrated in a graded ethanol series. Connective tissue was stained with Masson trichrome (Sigma, USA) following the protocol. Immunohistochemical staining of 8-hydroxydeoxyguanosine (8-OHdG) was performed following the protocol -(UltraSensitive^™^ S-P Kit, Maxim Bio, China). As negative controls for immunohistochemistry analysis, sections were incubated with nonimmune serum instead of the primary antibody and showed no staining. Images were then analyzed with Image-Pro Plus5.1.

### Statistical analyses

All statistical analyses were performed with SPSS 21.0 (IBM Corporation, Armonk, NY, USA), and data are presented as mean ± SD. Data were further subjected to analysis of variance (ANOVA). Differences between two groups were determined using Student’s *t* test, and multiple means were compared by Tukey’s test. *P* values < 0.05 were considered statistically significant.

## Results

### LPP decreased after VD and recovered over time

**Fig. 1:**
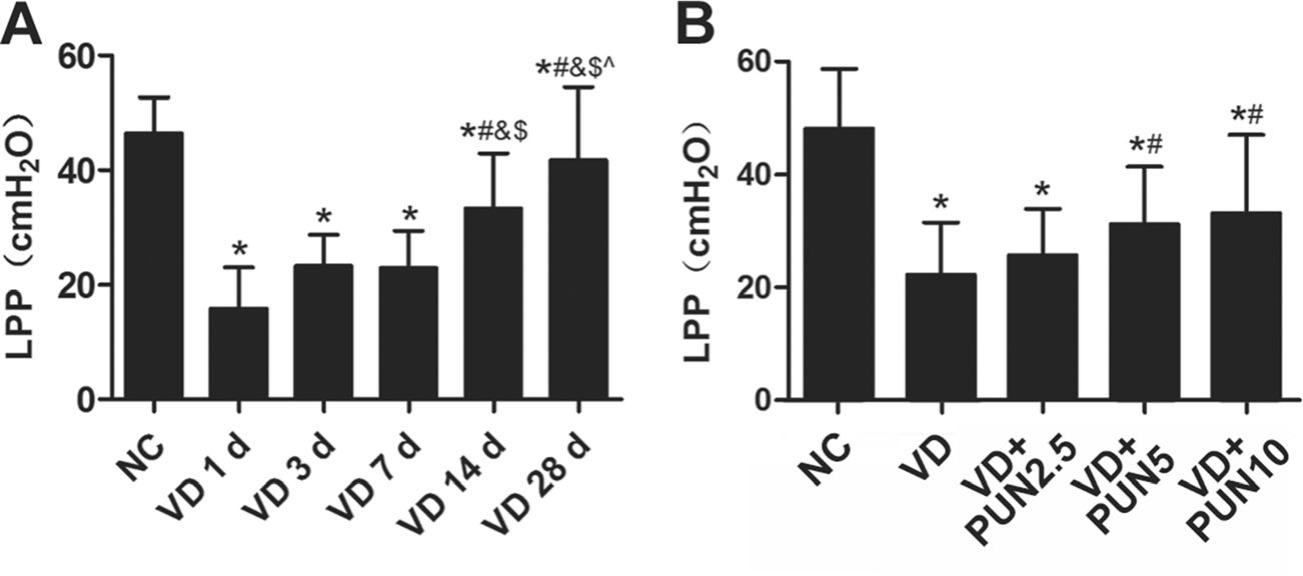
Leak-point pressure (LPP) values: **a** Mice in vaginal distention (VD) groups 1 d, 3 d, 7 d, 14 d, and 28 d were significantly lower than those in noninstrumented control (NC) group, and LPP recovered from the day 14 after VD. *P < 0.05 vs NC group; ^#^ *P* < 0.05 vs VD 1 d group; &*P* < 0.05 vs VD 3 d group; ^$^ *P* < 0.05 vs VD 7 d group; ^^^ *P* < 0.05 vs VD 14 d group. **b** LPP values of mice in VD, VD + punicalagin (PUN) 2.5, VD + PUN 5, and VD + PUN 10 groups were significantly lower than the NC group, whereas, values were significantly increased in VD + PUN 5 and VD + PUN 10 groups compared with the NC group. *P < 0.05 vs NC group; ^#^ *P* < 0.05 compared with VD group. Every measurement was repeated five times

The VD induced SUI mouse model shows self-restoring capacity. In this study, as show in Table 4 and Fig. 1a, all LPPs measured on days 1, 3, 7, 14, and 28 after VD were significantly decreased when compared with the NC group. No significant change was found on days 1, 3, and 7 after VD. However, on the day 14 after VD, LPP was significantly higher than on days 1, 3, and 7 and significantly lower than on day 28 after VD. Therefore, VD-induced SUI mouse model stabilized 7 days after VD and recovered from day 14.

**Table 4:** Leak-point pressure of mice in six groups $\left(\overline{x}\pm S\right)$

Group	LPP (cmH_2_O)	95% CI
NC	46.42 ± 6.30	(42.94–49.91)
VD 1 d	15.88 ± 7.07	(11.97–19.79)
VD 3 d	23.23 ± 5.46	(20.21–26.26)
VD 7 d	22.84 ± 6.58	(19.20–26.48)
VD 14 d	33.35 ± 9.60	(28.04–38.67)
VD 28 d	41.73 ± 12.73	(34.68–48.78)

*NC* noninstrumented control,* VD* vaginal distention,* LPP* leak-point pressure,* CI* confidence interval

### PUN promotes LPP recovery after VD in mice

Our preliminary result indicated that the VD-induced SUI mouse model was stable 7 days after VD. Hence, we tested LPPs of mice on day 7 after VD for more medication time. Table 5 and Fig. 1b show LPPs of mice in five groups. LPPs were significantly decreased in the VD and PUN 2.5, 5, and 10 groups compared with the NC group. Notably, treatment with 5 and 10 mg/kg PUN after VD significantly increased LPP; no significant change was found between PUN 2.5 and VD groups.

**Table 5:** Leak-point pressure (LPP) of mice in five groups $\left(\overline{x}\pm S\right)$

Group	LPP (cmH_2_O)	95% CI
NC	48.17 ± 10.52	(42.34–54.00)
VD	22.19 ± 9.33	(17.02–27.36)
VD + PUN 2.5	25.73 ± 8.16	(21.21–30.25)
VD + PUN 5	31.16 ± 10.25	(25.48–36.84)
VD + PUN 10	33.16 ± 13.88	(25.47–40.85)

*NC* noninstrumented control,* VD* vaginal distention,* PUN* punicalagin,* LPP* leak-point pressure,* CI* confidence interval

### PUN prevents VD-induced metabolic disorder of ECM in urethras and anterior vaginal wall of mice

**Fig. 2:**
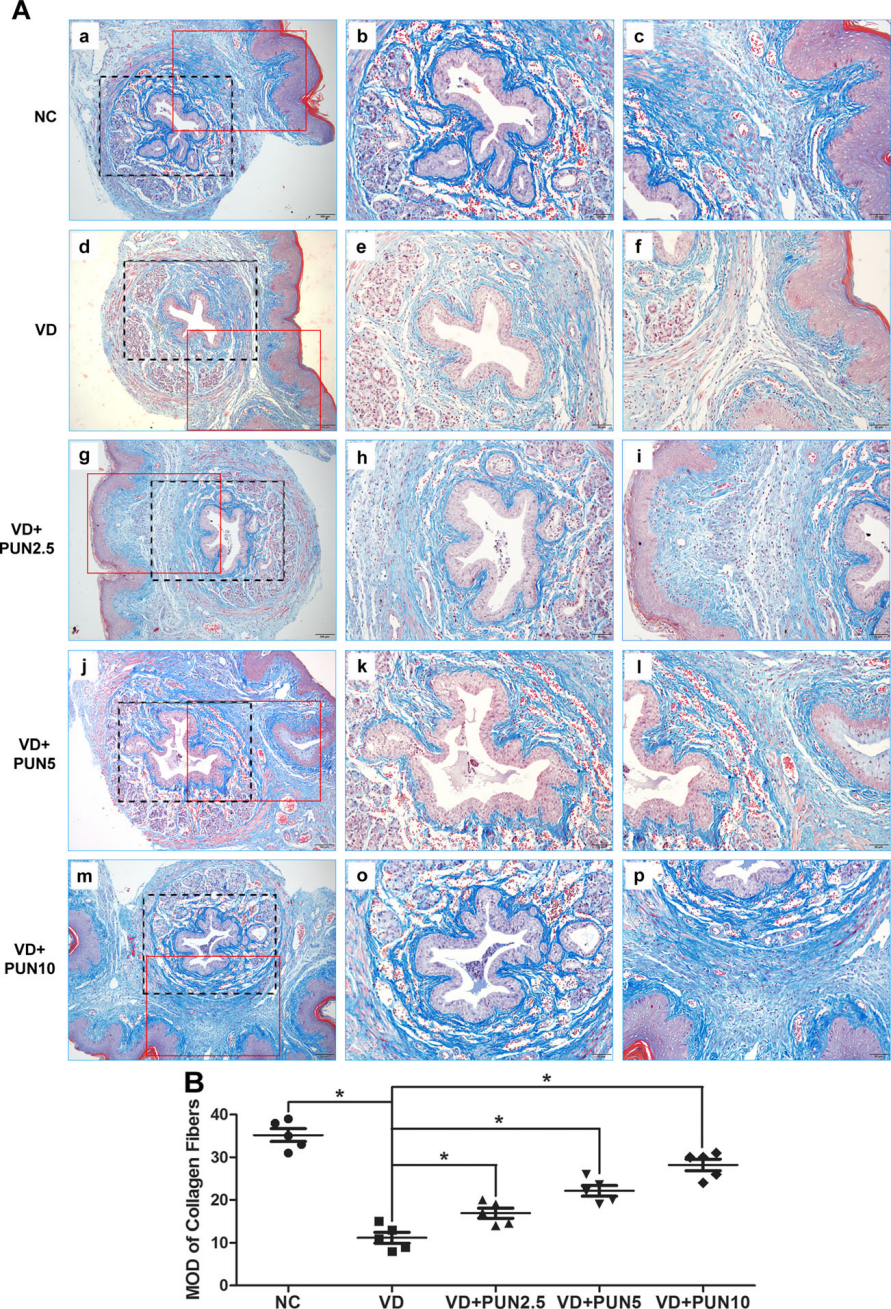
Masson trichrome staining of urethra and anterior vaginal wall: **a** noninstrumented control (NC) group (*a–c*), vaginal distention (VD) group (*d–f*), VD + punicalagin (PUN) 2.5 group (*g–i*), VD + PUN 5 group (*j–l*), and VD + PUN 10 group (*m–p*). Collagen fibers stained* blue*, muscle* white*, cellulose and cytoplasm* red*, nuclei* blue-purple*.* Second column*: magnification of the* black rectangle *in the first column;* third column*: magnification of the* red dotted rectangle *in the first column. Original magnification: ×100 (*a, d, g, j, m*); ×200 (*b–c, e–f, h–i, k–l, o–p*). **b** Semiquantitative assay of collagen using quantity one-4.6.2. *P < 0.05. Every experiment was repeated three times

**Fig. 3:**
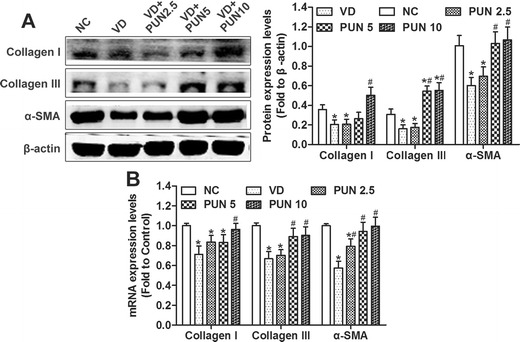
Protein and messenger RNA (mRNA) expressions of collagen I, collagen III, and α-smooth-muscle actin (SMA) in five groups. **a** Western blotting was performed to detect protein expression of collagen I, collagen III, and α-SMA in urethra and anterior vaginal wall of mice in five groups, and semiquantitative assay was done using quantity one-4.6.2. **b** Messenger RNA (mRNA) expression of collagen I, collagen III, and α-SMA in urethra and anterior vaginal wall of mice in five groups were detected by quantitative polymerase chain reaction (q-PCR). *P < 0.05 vs nonintrumented control (NC) group; ^#^ *P* < 0.05 compared with vaginal distention (VD) group; every experiment was repeated three times

Histologic examination of the midurethra showed typical morphology of urethra and anterior vaginal wall (Fig. 2a). Urethral muscle fibers were disrupted (Fig. 2a) and connective tissues decreased in the VD compared with the NC group (Fig. 2b). Connective tissues in urethra and anterior vaginal wall were apparently increased in a dose-dependent manner in mice in the VD + PUN group compared with the VD-alone group (Fig. 2b). In addition, the results of Western blot and q-PCR analysis (Fig. 3) show that both protein (Fig. 3a) and messenger RNA (mRNA) (Fig. 3b) expression levels of collagen I, collagen III, and α-SMA in urethra and anterior vaginal wall were significantly decreased after VD than those in the NC group. PUN increased protein expression of collagen I, collagen III, and α-SMA in a dose-dependent manner in VD + PUN mice compared with VD alone.

### PUN reverses VD-evoked TGFβ1/Smad3 signaling inhibition in urethras and anterior vaginal wall of mice

**Fig. 4:**
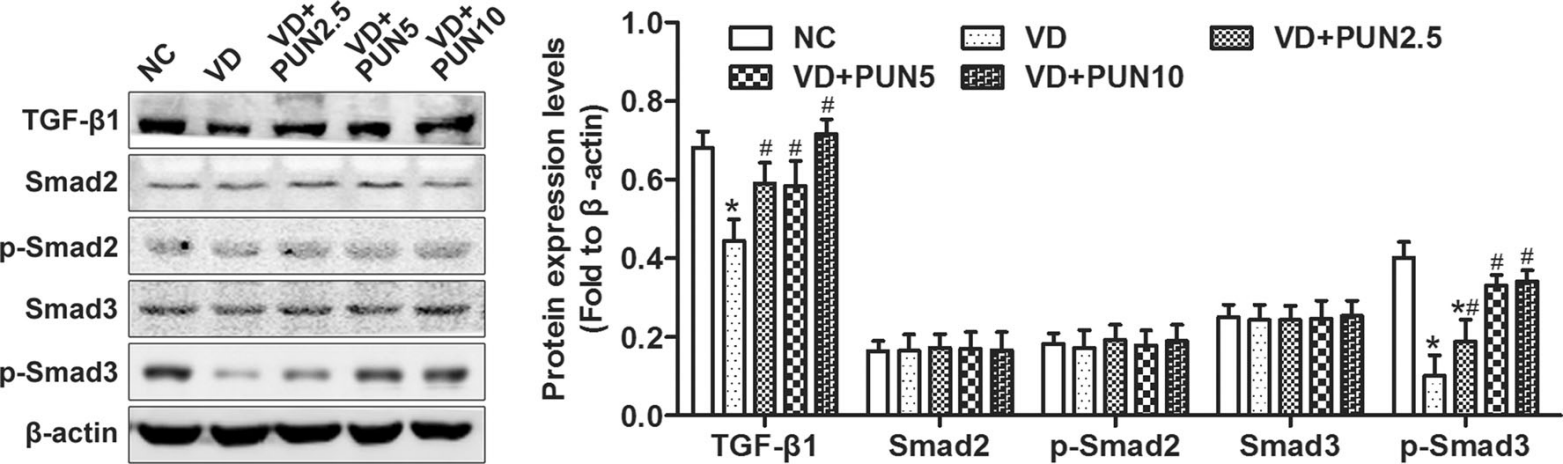
Western blotting shows protein expressions of transforming growth factor (TGF)-β1/Smads signaling pathways in urethra and anterior vaginal wall of mice in five groups. Semiquantitative assay was done using quantity one-4.6.2. *P < 0.05 compared with NC group; ^#^ *P* < 0.05 compared with VD group. Every experiment was repeated three times

TGF-β/Smads signaling plays an vital role in metabolism of ECM and has been reported to have participated in the pathological process of mechanical-trauma-induced SUI. In this study, results shown in Fig. 4 that expression levels of TGF-β1 and p-Smad3 were significantly decreased after VD compared with the NC group, but there were no significance changes in Smad2, p-Smad2, and Smad3. Similarly, PUN markedly increased TGF-β1 and p-Smad3 protein expression in a dose-dependent manner in the urethra and anterior vaginal wall after VD than in the NC group, with no significant effect on protein expression of Smad2, p-Smad2, and Smad3.

### PUN alleviates VD-induced oxidative damage in urethras and anterior vaginal wall of mice through upregulation of Nrf2/ARE signaling

**Fig. 5:**
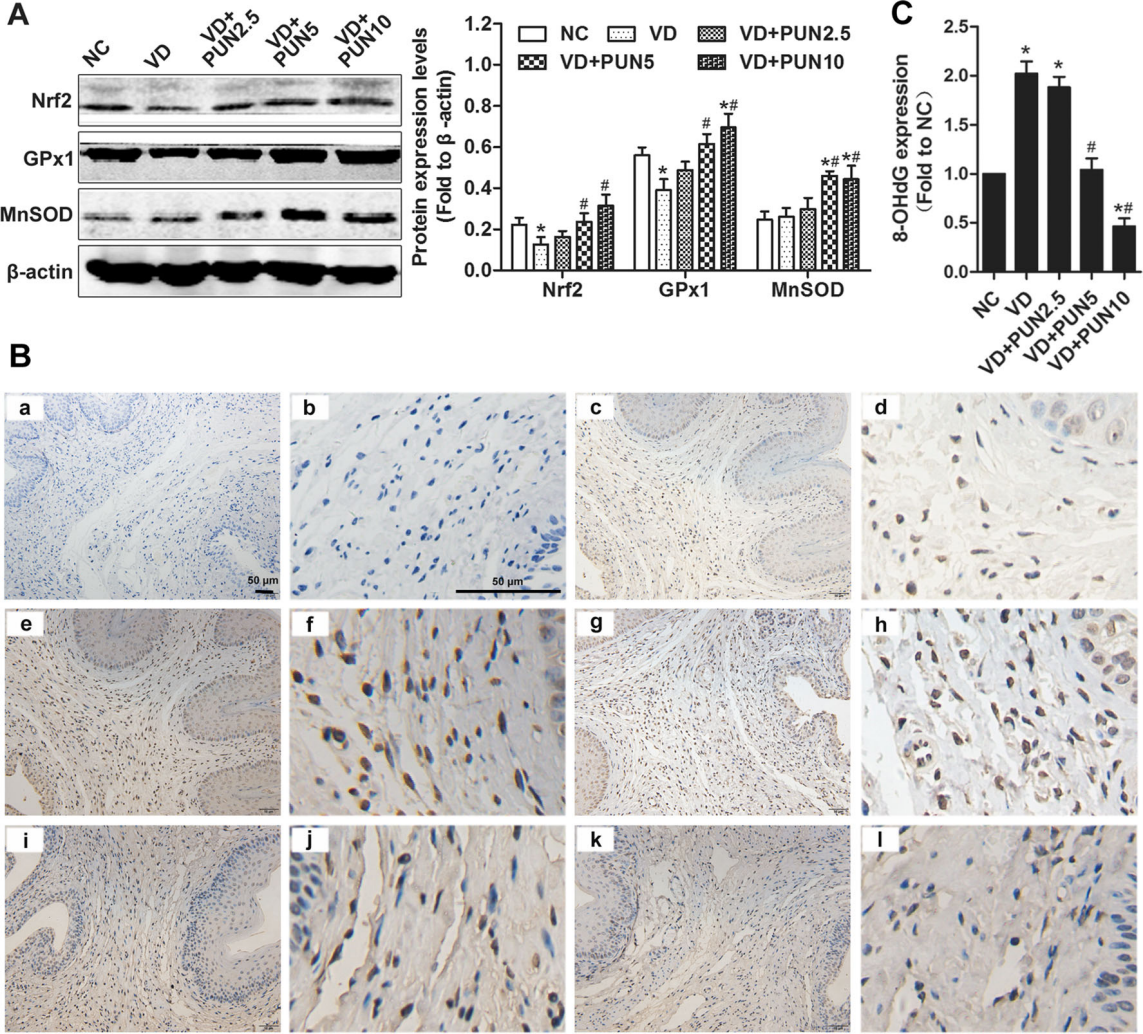
Nuclear factor-erythroid 2 p45-related factor 2 (Nrf2)/antioxidant response element (ARE) signaling-related proteins and 8-hydroxydeoxyguanosine (8-OHdG) expression in five groups. **a** Western blotting detected Nrf2, glutathione peroxidase (GPx1), and manganese superoxide dismutase (MnSOD) in urethra and anterior vaginal wall of mice in five groups. Semiquantitative assay was done using quantity one-4.6.2. **b** Expression of 8-OHdG was detected using immunohistochemistry in urethra and anterior vaginal wall; representative images are shown: negative control (*a–b*); noninstrumented control (NC) (*c–d*); vaginal dilation (VD) (*e–f*2); VD + punicalagin (PUN) 2.5 (*g–h*); VD + PUN 5 (*i–j*); VD + PUN 10 (*k–l*). Images in the* second column* are magnification of the first column; the images in the* forth column* are magnification of the third column. Scale = 50 μm. **c** Semiquantitative assay used Image-Pro Plus 5.1. *P < 0.05 cvs ^#^ *P* < 0.05 vs VD. Every experiment was repeated three times

To identify the involvement of Nrf2/ARE signaling activation in PUN against mechanical-trauma-induced SUI, we detected protein expression of Nrf2, GPx1, MnSOD, and the oxidative damage biomarker 8-OHdG in mice. As shown in Fig. 5a, expression levels of Nrf2 and GPx1 in urethra and anterior vaginal wall were significantly decreased after VD than in the NC group, and there was no significant difference in MnSOD groups. In addition, PUN increased Nrf2, GPx1, and MnSOD expression in a dose-dependent manner, and 8-OHdG expression was significantly increased in the VD group (Fig. 5b–c). However, 8-OHdG in PUN groups 5 and 10 were significantly decreased compared with the VD group (Fig. 5b–c).

## Discussion

SUI is a common social and hygiene problem affecting the QoL of women worldwide and causing serious social economic load. Mechanical trauma, such as connective tissue, muscle, or nerve, or damage to the vaginal wall or urethra and its suspensory structures due to VD is a widely recognized risk factor in the genesis of SUI. In addition, there are as yet no drugs to effectively cure SUI. In this study, we used PUN to reverse mechanical-trauma-induced SUI in a mouse model and found it has a therapeutic role against mechanical-trauma-induced decreases in LPP and metabolic disorders of the ECM. This effect may be via TGF-β1/Smad3, and Nrf2/ARE signaling activation.

VD is used to induce SUI in mice and rats, as evidenced by LPP and/or maximal urethral closing pressure (MUCP) on urodynamic testing [1, 8, 21, 22]. An SUI mouse model revealed that a 0.3-ml intravaginal balloon (diameter ∼8 mm, equal to brain-case diameter of newborn mice) produced greater LPP than 0.1- and 0.2-ml balloons [23]. VD-induced SUI mouse model possesses self-restoring capacity [24, 25], and LPP on measurements on days 0, 4, 10, and 20 after VD showed that LPP was restored from day 20 [25]. 

In our study reported here, we measured LPP on days 1, 3, 7, 14, and 28 after VD. Seven days after VD, LPP was markedly decreased from that of the NC group and began to recover from day 14. Based on this result, we tested LPPs on day 7 following VD and PUN oral therapy. We found that PUN has the potential to cure mechanical-trauma-induced SUI in mice and report such findings here for the first time.

The vesical neck and urethra are attached to the anterior vaginal wall, which has fascial connections to the levator ani muscles through the arcus tendineus fasciae pelvis. In addition, connective tissue comprising collagen and elastin, as well as muscle tissue, provides the vaginal wall with sufficient strength and resilience to maintain normal anatomic position and function. Pelvic floor injury (such as during childbirth) may disrupt these supportive tissues and connections, causing the urethra to lose its hammock-like support and resulting in SUI [26]. In this study, urethral muscle fibers were disrupted, connective tissue strength decreased, and both protein and mRNA expressions of collagen I, collagen III, and α-SMA decreased in urethra and the anterior vaginal wall after VD. However, PUN significantly alleviated the metabolic disorder of ECM. This may be due to the mechanism of PUN against mechanical-trauma-induced SUI.

TGF-β1 signaling plays an important role in collagen and α-SMA regulation.Studies show that TGF-β1 signaling is involved in the pathological process of mechanical-trauma-induced SUI [27–29]. In addition, TGF-β1 expression in uterosacral ligaments of women with POP and SUI was decreased compared with women with POP only or in the control group [15]. To determine whether TGF-β1 signaling is involved in PUN-induced increase in ECM after VD, we assessed changes in TGF-β1 signaling in the mouse urethra and anterior vaginal. Our results show that the protein expression levels of TGF-β1 and p-Smad3 in the VD group were significantly down-regulated compared with the NC group, with no significant difference in Smad2, p-Smad2, and Smad3 expression, whereas PUN activated VD-induced TGF-β1/Smad3 signaling inhibition. One study indicated that TGF-β1 and p-Smad2 expression was up-regulated in urethral tissue of SUI rats and not in the sham group [29]. Another study found that Smad7 expression decreased and Smad3 increased in urethral tissue of SUI rats vs the control group [28]. However, those studies measured TGF-β signaling 4 weeks after VD; our results show that the LPP began to recover from day 14. We tested TGF-β signaling on day 7 also, which showed early-phase changes in TGF-β signaling in both urethra and anterior vaginal wall of our mouse model, whereas the rat models reflect the later-phase changes in urethral tissues. Hence, we deduce that TGF-β signaling was inhibited in the early phase and activated in the later phase of our study. In addition, changes in Smad3 were antecedent to Smad2. As a result, LPP was decreased in the early phase and gradually recovered in the later phase of recovery from VD. As a consequence, TGF-β signaling may be an important target for preventing and treating SUI.

Oxidative damage to vaginal wall and urethral sphincter was confirmed as a result of mechanical trauma and was prominent in the pathological process of pelvic floor dysfunction (PFD) [7–11]. Previous study indicated that cyclic mechanical strain increased oxidative damage to human parametrial ligament fibroblasts and may be a pathogenesis of PFD [30]. A recent study showed that H_2_O_2_ significantly decreased the contractile force of isolated strips of bladder and bladder-base urethra [11]. PUN, as a type of antioxidant, has shown to alleviate oxidative damage both in vivo and in vitro by upregulating Nrf2/ARE signaling pathway. 

In this study, protein expressions of Nrf2 and its downstream GPx1 were significantly decreased in VD mice, and oxidative damage aggravated the urethra and anterior vaginal wall. However, PUN up-regulated expressions of antioxidant proteins Nrf2 and GPx1 and alleviated VD-induced oxidative damage to the urethra and anterior vaginal wall. Hence, mechanical-trauma-induced decrease in antioxidant capacity and increase in oxidative damage to urethra and anterior vaginal wall may be the pathogenesis of a metabolic disorder of the ECM and result in SUI. Therefore, the oxidation–antioxidation system may be an important target for SUI prevention and treatment. Some antioxidant foods or drugs may have beneficial protective effects against mechanical-trauma-induced SUI.

In conclusion, our results suggest that mechanical-trauma-induced VD can cause SUI in female mice, recovery begins at day 14. In the early phase after VD, direct disruption of the urethral structure and TGF-β1/Smad3 and Nrf2/ARE signaling inhibition induced a metabolic disorder in the ECM. . PUN had certain therapeutic effects on mechanical-trauma-induced SUI by up-regulating Nrf2/ARE and TGF-β1/Smad3 pathways. However, further research is needed to confirm the relationship between these two signaling processes in the pathogenesis of mechanical-trauma-induced SUI.
